# Perioperative chemotherapy versus neoadjuvant chemoradiation for patients with adenocarcinoma of the distal esophagus in Austria: a retrospective analysis

**DOI:** 10.1186/s12957-019-1693-6

**Published:** 2019-08-19

**Authors:** Oliver O. Koch, Michael Weitzendorfer, Martin Varga, Andreas Tschoner, Richard Partl, Alexander Perathoner, Philipp Gehwolf, Karin S. Kapp, Reinhold Függer, Dietmar Öfner, Klaus Emmanuel

**Affiliations:** 1 Department of Visceral and Thoracic Surgery, Paracelsus Medical University, Salzburg, Austria; 2 Department of General and Visceral Surgery, Ordensklinikum Linz Barmherzige Schwestern, Linz, Austria; 3 Department of Therapeutic Radiology and Oncology, Comprehensive Cancer Center, Medical University of Graz, Graz, Austria; 4 Department of Visceral, Transplant and Thoracic Surgery, Medical University of Innsbruck, Innsbruck, Austria

**Keywords:** Adenocarcinoma, Esophagus, Chemotherapy, Chemoradiation, Esophagectomy

## Abstract

**Background:**

The aim of this study was to compare the outcome of patients with adenocarcinoma of the distal esophagus (AEG type I) treated with neoadjuvant chemoradiation or perioperative chemotherapy.

**Methods:**

Eligible patients from four Austrian centers were selected to conduct a retrospective analysis. All patients treated between January 2007 and October 2017 with chemotherapy according to EOX-protocol (Epirubicin, Oxaliplatin, Xeloda) or chemoradiation according to CROSS-protocol (carboplatin/paclitaxel + RTX 41.4 Gy), before esophagectomy were included. Primary outcomes disease-free survival (DFS) and overall survival (OS) as well as secondary outcomes downstaging of T- or N-stage and achievement of pathological complete response pCR (ypT0N0M0) were analyzed. Data of 119 patients were included.

**Results:**

Complete data was available in 104 patients, 53 patients in the chemoradiation group and 51 patients in the chemotherapy group. The mean number of lymph nodes removed was significantly higher in the EOX group (EOX 29 ± 15.5 vs. CROSS 22 ± 8.8; *p* < 0.05). Median follow-up in the CROSS group was 17 months (CI 95% 8.8–25.2) and in the EOX group 37 months (CI 95% 26.5–47.5).

In the chemotherapy group, the OS rate after half a year, − 1, and 3 years was 92%, 75%, and 51%. After chemoradiation, overall survival after half a year was 85 %, after 1 year 66%, and after 3 years 17%. In the EOX group DFS after ½, − 1, and 3 years was 90%, 73%, and 45%, in the chemoradiation group after half a year 81%, after 1 year 55% and after 3 years 15%. Pathological complete response (pCR) was achieved in 23% of patients after CROSS and in 10% after EOX (*p* < 0.000).

**Conclusions:**

There seem to be clear advantages for chemoradiation, concerning the major response of the primary tumor, whereas a tendency in favor for chemotherapy is seen in regards to systemic tumor control. Furthermore, the type of neoadjuvant treatment has a significant influence on the number of lymph nodes resected.

## Introduction

In Austria, each year approximately 400 people are newly diagnosed with esophageal cancer [1]. The incidence of esophageal carcinomas is continuing to rise worldwide. A recent analysis of 43 cancer registries of Europe, Canada, the USA, and Australia showed that the incidence of adenocarcinoma of the esophagus has been increasing rapidly in the past two decades [2].

Management of gastro-esophageal junction (GOJ) adenocarcinoma is still a controversial question. The actual recommendation refers to the Siewert classification, which is based on the tumor location. An adenocarcinoma of the distal esophagus is classified as AEG type I, adenocarcinoma of the gastroesophageal junction = AEG type II and proximal stomach = AEG type III [3].

Surgery is the cornerstone of treatment of esophageal adenocarcinoma, but survival after resection alone is weak. Several studies could show that outcomes improve if chemotherapy or chemoradiotherapy is part of the treatment, and therefore a multimodal approach should be the standard for locally advanced tumors [4]. However, especially in case of AEG type I which type of neo-adjuvant treatment is under debate, because both neoadjuvant chemoradiotherapy and perioperative chemotherapy have shown an improvement of survival rates over surgery alone [5–8].

The majority of scientific evidence for adenocarcinoma of the distal esophagus is from studies designed for esophagus or stomach tumors, and therefore not quite sufficient. The MAGIC-trial and the ACCORD-Trial showed a clear survival benefit when chemotherapy was added in perioperative fashion over surgery alone [5, 9].

Several following studies proved that oral capecitabine is as effective as fluorouracil in patients with this type of cancer [10, 11] and newer protocols like FLOT (5-fluorouracil/leucovorin, oxaliplatin, and docetaxel) showed an even better response [12–14].

In the intention to improve locoregional tumor control and the complete resection rate, the adding of radiotherapy has also been studied. The landmark study in this concern was the Dutch CROSS-trial published in 2012 comparing surgery alone to surgery after neoadjuvant radiochemotherapy in patients with squamous cell carcinoma and adenocarcinoma. The trial showed a greater R0 resection rate and better global survival after in the neoadjuvant treatment (49.4 months vs. 24.0 months) [15].

The benefits showed up to be clinically relevant for both adenocarcinoma, squamous cell carcinoma, and since the adverse events of the CROSS protocol are low too, radiochemotherapy followed by surgery has become the standard of care in many centers.

Advantages and disadvantages are seen in each neoadjuvant treatment regimen. Radiochemotherapy has good loco-regional control but maybe lesser control than chemotherapy on systemic metastasis. Since data comparing these two treatment options with each other is scarce, the choice between the two is still under discussion. The aim of this study was to compare the outcome of patients with AEG type I carcinoma treated with neoadjuvant chemoradiation or perioperative chemotherapy.

## Materials and methods

### Patients

A retrospective analysis of eligible patients from four Austrian centers was conducted to find out which pre/perioperative therapy has a better outcome for patients with a resectable AEG type I tumor. The four centers were

- Department of Visceral and Thoracic Surgery, Paracelsus Medical University, Salzburg, Austria
- Department of Visceral, Transplant and Thoracic Surgery, Medical University of Innsbruck, Innsbruck, Austria
- Department of Therapeutic Radiology and Oncology, Comprehensive Cancer Center Graz, Medical University of Graz, Austria and
- Department of General and Visceral Surgery, Ordensklinikum Linz Barmherzige Schwestern, Linz, Austria.

From the databases of these four centers, all patients with AEG type I treated between January 2007 and July 2017 with chemotherapy according to EOX-Protocol (epirubicin, oxaliplatin, xeloda) or chemoradiation according to CROSS-Protocol (carboplatin/paclitaxel + RTX 41.4 Gy), followed by curative esophagectomy (defined as R0) were included in the study.

Clinical staging in all patients was performed by endoscopy with biopsy, endoscopic ultrasonography, and either standalone computed tomography (CT) or integrated 18F-fluorodeoxyglucose positron emission tomography (18F-FDG PET)/CT scanning. Restaging consisted of the same scheme after neoadjuvant treatment to exclude patients with tumor progression. All patients had a biopsy-proven resectable adenocarcinoma of the distal esophagus (clinical stage T1N1-3 or T2-4aN0-3) without distant metastases. Study approval was obtained according to the ethics committee of the state of Salzburg.

### Therapy

Neoadjuvant treatment protocols were administrated according to CROSS or EOX.

The CROSS protocol consisted of the preoperative total radiation dose of 41.4 Gy. The patients received 1.8 Gy in 23 fractions within 5 weeks and weekly administration of carboplatin (targeted at an area under the curve of 2 mg/mL per min) and paclitaxel (50 mg/m^2^ of body-surface area) [15].

In the chemotherapy regime patients received pre- and postoperative epirubicin (50 mg/m^2^) and oxaliplatin (130 mg/m^2^) in 3-week cycles of an intravenous bolus, followed by 625 mg/m^2^ of capecitabine twice daily for 21 days.

After neoadjuvant treatment, a transthoracic esophagectomy with en bloc two-field lymphadenectomy was performed in all patients, followed by gastric conduit. Reconstruction was either with intrathoracic anastomosis or cervical anastomosis according to the preference of the different centers.

### Data collection and follow-up

Follow-up was similar in all centers and included physical examination, patient history, endoscopy, plain chest radiography, tumor marker, abdominal ultrasound, CT scans of the abdomen and chest, and PET-CT scans. It was performed in a 3-month interval during year 1, 6 months interval during year number 2, and annually from the third year on. All data were collected from the hospital databases of the centers. Recurrence was confirmed by histology or by clinical follow-up. Progression of disease was defined as either local recurrence or as metastases in distant organs or juxtaregional nodes. Disease-free survival (DFS) and overall survival (OS) were calculated from the time of the first diagnosis until recurrence or last follow-up, or the date of death or last follow-up, respectively.

### Histopatholgy

All specimens were analyzed by specialized gastrointestinal pathologists using a standardized protocol in accordance with the current edition of the International Union Against Cancer for ypTNM-classification [16].

### Statistical analysis

Statistical analysis was performed using the Statistical Package for Social Sciences (SPSS) computer program (SPSS Inc. Version 19, Chicago, IL, USA). Data are presented as means ± standard deviation, range, or percentage.

Survival distribution was described by Kaplan-Meier plots and the survival differences were evaluated by log-rank test. In addition, mean and median survival rates were calculated with 95% confidence intervals (CIs) reported. A *p* value below 0.05 was considered statistically significant.

## Results

A total of 119 patients with AEG I received preoperative therapy with CROSS or EOX followed by curative esophagectomy. Complete data was available in 104 patients, 53 patients in the chemoradiation group and 51 patients in the chemotherapy group.

No significant difference in demographic data was found between the two groups. However, a significant difference in T-stage but not in N-stage between the two groups was found (Table 1).

**Table 1:** Demographic data and tumor-stage

Patients demographics and clinical characteristics	CROSS *n* = 53	EOX *n* = 51	*p* value
Age, year ^a^	60.2 ± 9.2	60.1 ± 12.3	0.975
Male gender	48 (90.6)	45 (88.2)	0.703
cT stage^b^			0.023
T1	0 (0.0)	3 (6.8)	
T2	5 (11.9)	8 (18.2)	
T3	37 (88.1)	29 (65.9)	
T4	0 (0.0)	4 (9.1)	
cN-stage ^c^			0.884
N0	12 (30.8)	12 (29.3)	
N+	27 (69.2)	29 (70.7)	

Data are numbers of patients with percentages in parentheses

^a^Data are mean ± standard deviation

^b^Clinical tumor stage (cT) classified according to the 8th edition of the International Union Against Cancer (UICC) tumor-node-metastasis (TNM) classification [16]

^c^Clinical lymph node (cN) stage classified according to the 8th edition of the UICC TNM classification [16]

### Preoperative course

In the chemotherapy group, 42/51 (82%) patients received the complete treatment regimen. The main reasons for not completing all 3 chemotherapy cycles were adverse effects of the therapy (acute renal failure, reduced general condition, thrombosis, and cytopenia). Seven patients received 2 and 2 patients 1 cycle of chemotherapy.

In the chemoradiation group, 43/53 (81%) patients received all 5 cycles of chemotherapy. Six patients received 4 and 4 patients 3 cycles of chemotherapy. For not completing all chemotherapy cycles, hematologic adverse effect with cytopenia were the main reasons. The initially planned radiation dose was achieved in all patients.

### Postoperative course

All patients included in the study underwent surgical R0 resection. No cases of 30-day postoperative mortality were described. The 90-day postoperative mortality rate was 1.9% (1/51) for the EOX-group; in the CROSS-group, there were no cases. Minor postoperative complications were not documented in all of the participating centers. Severe complications like anastomotic leakage, pneumonia, cardiac arrhythmia, and chyle leak were documented in all of the centers. The incidences of these complications were comparable between both groups (Table 2). In total 36/104 patients had severe postoperative complications (34.6%). The most common complications were anastomotic leakage (20.3%), pneumonia (7.7%), and chyle leak (4.8%).

**Table 2:** Comparative analysis of postoperative course

	CROSS *n* = 53	EOX *n* = 51	*p* value
Complicated postoperative course	17 (32.1)	19 (37.3)	0.781
Anastomotic leakage^a^	9 (17.0)	12 (23.5)	0.807
Pneumonia^b^	4 (7.5)	4 (7.8)	0.920
Chyle leak^c^	3 (5.7)	2 (3.9)	0.888
Cardiac arrythmia^d^	1 (1.9)	1 (2.0)	1.000

Data are numbers of patients with percentages in parentheses

^a^Anastomotic leakage included all clinical and radiological findings of anastomotic dehiscence or fistula

^b^Pneumonia was defined by the universal pneumonia score [17]

^c^Chyle leak was defined as elevated levels of triglycerides in intrathoracic fluid requiring treatment

^d^Cardiac arrhythmia were defined as any change in rhythm on an electrocardiogram requiring treatment

Adjuvant chemotherapy after surgery was started in 23/51 (45.1%) of the patients in the chemotherapy group. The reasons why the patients did not start the intended postoperative therapy were not documented in all of the centers. Of the 23 patients who started with the postoperative chemotherapy, 1 patient received only one postoperative cycle, two patients received 2 cycles of chemotherapy, and 20/51 (39%) received all postoperative cycles of chemotherapy. No statistical differences occurred in preoperative tumor characteristics and patient-related characteristics between patients who did or did not undergo postoperative chemotherapy.

### Survival

**Fig. 1:**
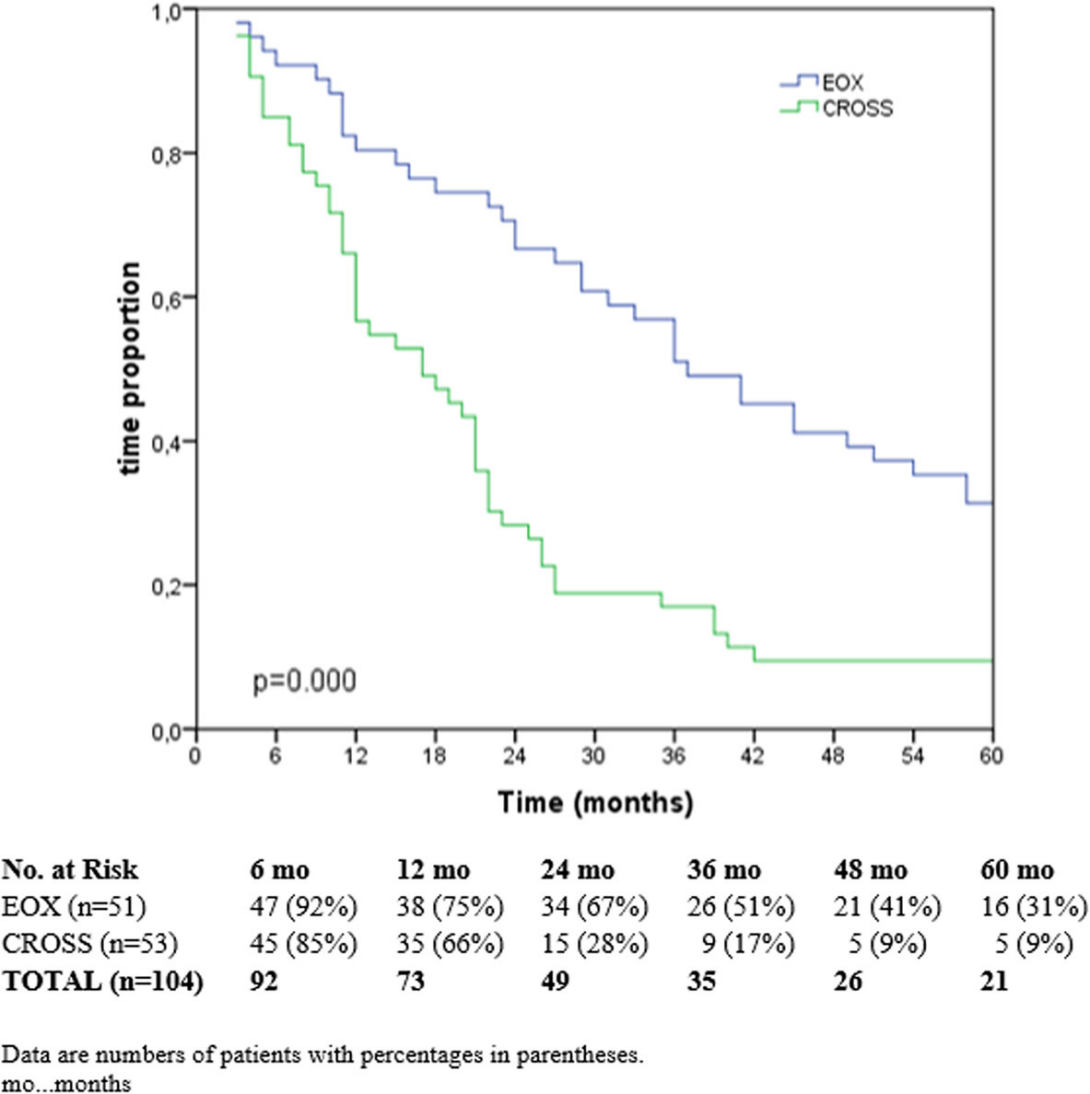
Overall survival CROSS vs. EOX

The median follow-up time was 17.0 months (CI 95% 8.8–25.2) in the CROSS group and 37.0 months (CI 95% 26.5–47.5) in the EOX group. Significant differences in OS and DFS was found between the two therapies. Overall survival rates in the chemotherapy group after half a year, 1, 3, and 5 years were 92%, 75%, 51%, and 31%, respectively. Follow-up data from the CROSS group comprised only half and 1 year. Respectively, overall survival was 85% and 66%. OS was significantly better in the EOX group (*p* < 0.000) (Fig. 1).

**Fig. 2:**
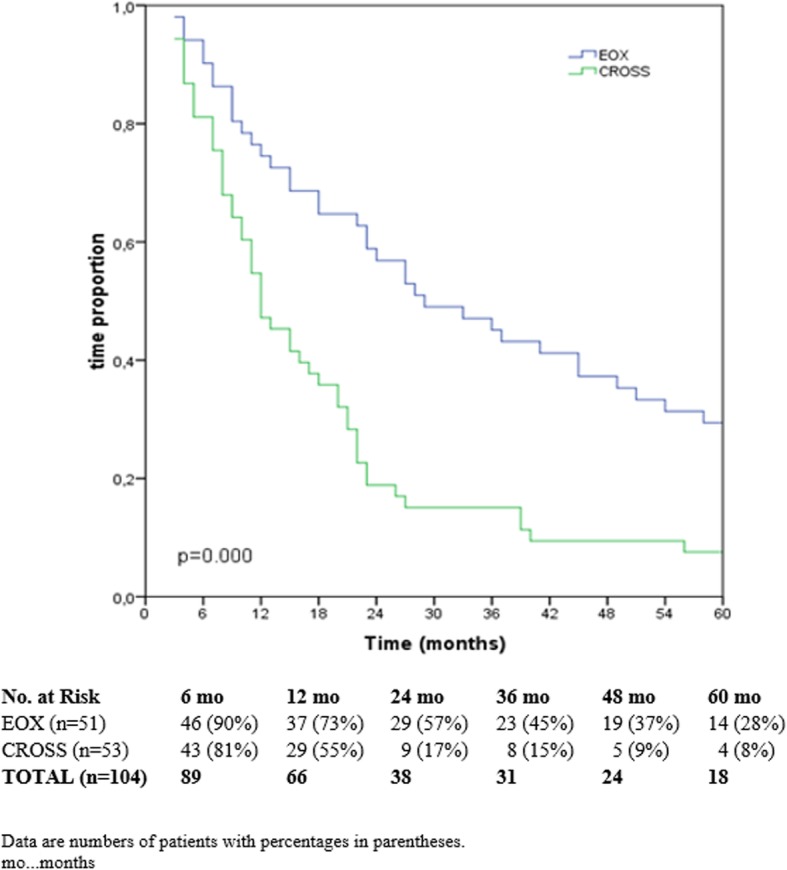
Disease-free survival CROSS vs. EOX

Disease-free survival rates in the EOX group after half a year, 1, 3, and 5 years were 90%, 73%, 45%, and 28%, respectively. In the CROSS group, disease-free survival rates after half a year and 1 year were 81% and 55%. Disease-free survival was significantly better after EOX (*p* < 0.000) (Fig. 2).

### Histopathology

Since the study inclusion criteria were R0 resection, all patients analyzed received curative esophagectomy. Tumor response to preoperative therapy was analyzed by comparing preoperative radiological TNM staging with the histopathological findings after surgical resection.

A pathological complete response (pCR) with pT0N0 in histological finding was achieved in 17 of 104 patients (16%). A significant difference was found in pCR rate in patients who underwent radiochemotherapy compared to perioperative chemotherapy (12 vs. 5 patients, 23% vs. 10% respectively, *p* = 0.000).

Downstaging of T and N status was compared between both groups. Down staging of T status was achieved in the CROSS group 21 times vs. 14 times in EOX group (*p* > 0.05), and 10 vs. 15 times in N status (*p* > 0.05). Simultaneous downstaging of T and N status was achieved 9 times in both groups, what is without statistically significant difference.

Radiological staging and histopathological findings of complete response (pCR) and downstaging are shown in Table 3.

**Table 3:** Radiological staging and histopathological findings of complete response (pCR) and down staging

	CROSS *n* = 53	EOX *n* = 51	*p* value
ypT category^a^			0.165
T0	12 (25.0)	5 (10.6)	
T1	8 (16.7)	6 (12.8)	
T2	7 (14.6)	12 (25.5)	
T3	21 (43.8)	22 (46.8)	
T4	0 (0.0)	2 (4.3)	
ypN category ^b^			0.837
N0	23 (48.9)	25 (53.2)	
N+	24 (51.1)	22 (46.8)	
Downstaging			
T category	21 (39.6)	14 (27.5)	0.462
N category	10 (18.9)	15 (29.4)	0.517
T and N-category	9 (17.0)	9 (17.6)	0.982
pCR	12 (22.6)	5 (9.8)	< 0.001

*pCR* histopathological complete response

Data are numbers of patients with percentages in parentheses

^a^Pathological tumor stage (pT) classified according to the 8th edition of the International Union Against Cancer (UICC) tumor-node-metastasis (TNM) classification [16]

^b^Pathological lymph node (pN) stage classified according to the 8th edition of the UICC TNM classification [16]

Between the two groups, a significant difference in the number of resected lymph nodes therapies could be found (*p* < 0.05). After CROSS therapy, the mean number of resected lymph nodes was 22 (SD ± 8.8; range 5–38) vs 29 (SD ± 15.5; range 1–58) after EOX. No significant difference was found in the number of resected affected lymph nodes. The average number of affected lymph nodes after CROSS was 3 (SD ± 3.8) and after EOX also 3 (SD ± 4.7). The maximum number of resected affected lymph nodes was 14 (range 0–14), and in 16 patients no lymph nodes were affected after CROSS therapy. After EOX therapy, the maximum number of resected affected lymph nodes was 28 (range 0–28), and in 25 patients no lymph nodes were affected. Histopathological findings of the lymph nodes are presented in Table 4.

**Table 4:** Histopathological findings of the lymph nodes

	CROSS *n* = 53	EOX *n* = 51	*p* value
Lymph node counts ^a^			
Total LNs (range)	22 (5-38)	29 (1–58)	0.020
Metastatic LNs (range)	3 (0-14)	3 (0-28)	0.707
Not affected LNs^b^	16 (30.1%)	25 (49.0%)	0.269

^a^Data are mean ± standard deviation

^b^number of patients without affected lymph nodes

## Discussion

It is a matter of fact that patients with adenocarcinoma of the distal esophagus benefit from a multimodal concept of therapy. However which kind of neoadjuvant treatment is still under debate. A variety of different kinds of modalities have been tried in the past, but survival rates are still dissatisfying. In Austria, perioperative chemotherapy according to EOX protocol for AEG was very popular, but since the first reported results of the CROSS-trial neoadjuvant radiochemotherapy according to CROSS has gained popularity [15].

Radiochemotherapy has good loco-regional control but maybe lesser control than chemotherapy on systemic metastasis. Due to available data, the choice between the two treatment options is still equivocal. Results from prospective trials comparing the two neoadjuvant treatment options with each other in patients with AEG would be of crucial importance.

Recently, the results of the Scandinavian NeoRes I trial have been reported. Neoadjuvant chemoradiotherapy (40 Gy) was compared with neoadjuvant chemotherapy (cisplatin/fluorouracil) in this randomized phase II trial with no survival advantages were seen, despite a higher tumor tissue response in the chemoradiotherapy group. Patients included in the trial had squamous cell carcinoma or AEG I-II. In multivariate analysis, neither patients with adenocarcinoma nor patients with squamous cell carcinoma seemed to benefit from the addition of radiotherapy. n fact, the outcome of patients with adenocarcinoma being treated with neoadjuvant chemoradiotherapy was even slightly worse (*p* < 0.70). Accordingly, the authors concluded that these results from to date the largest completed randomized trial do not support the unselected addition of radiotherapy to neoadjuvant chemotherapy as a standard of care in esophageal cancer patients [18]. Noteworthy, cisplatin/fluorouracil chemotherapy in the trial was only given neoadjuvant. Meanwhile, we know that survival using perioperative chemotherapy protocols for patients with adenocarcinoma leads to even better outcomes [19].

However, the results of the NeoRes trial seem to validate the results of our trial, showing an inferior survival outcome for patients with AEG I receiving neoadjuvant radiochemotherapy compared to perioperative chemotherapy.

The fact that we permanently gain new knowledge which chemotherapy would be better had also influenced the ongoing AEGIS trial. The AEGIS trial of the Irish Clinical Research Group (ICORG) was originally designed comparing EOX with CROSS, but since the results of the FLOT4 trial were presented, showing the clear advantages of FLOT compared to EOX, the lead investigators changed their study protocol. In the Neo-AEGIS trial, the participating centers have the option between EOX or FLOT as chemotherapy treatment [19, 20]. The Neo-AEGIS is still recruiting patients, just as the German ESOPEC trial which also compares FLOT vs CROSS in patients with adenocarcinoma of the esophagus (NCT02509286) [21].

The results of these trials may be eagerly awaited until then we have to content ourselves with the results of retrospective analysis. However, the results of these retrospective analyses performed are contradictory and the perioperative chemotherapies and neoadjuvant chemoradiation regimes used were various. Hoeppner J. et al. were the first to analyze the outcome of perioperative chemotherapy vs neoadjuvant chemoradiation in 105 patients with locally advanced esophageal adenocarcinoma. The study showed a higher rate of histologic response to neoadjuvant chemoradiotherapy compared to perioperative chemotherapy, but without showing higher OS rates. Three and 5-year survival rates were significantly better after perioperative chemotherapy compared to neoadjuvant radiochemotherapy (52%/45% for neoadjuvant radiochemotherapy and 68%/63% for perioperative chemotherapy). Furthermore, perioperative chemotherapy showed fewer incidences of treatment-related morbidity and mortality. However, three different perioperative chemotherapy protocols were included in the study ECF, FLOT, and XELOX, and also the dose of radiotherapy in the radiochemotherapy group was not homogenous (45 Gy or 36 Gy).

In a recent Dutch retrospective analysis of patients who underwent surgery with esophageal or gastroesophageal junction adenocarcinoma, no significant differences regarding postoperative mortality and morbidity between patients who had perioperative chemotherapy (epirubicin, cisplatin, and capecitabine) or neoadjuvant radiochemotherapy according to CROSS, were seen. Moreover, and in contrast to the previous German study, no significant differences were found in 3-year progression-free survival (radiochemotherapy vs. chemotherapy 55% vs. 46%, *p* = 0.344) and overall survival rates (50% vs. 49%, *p* = 0.934) between the two therapies [22].

Recently, a third single-center retrospective trial was published. Locally advanced AEG type I or II carcinomas, treated with chemoradiation (CROSS-protocol) or, chemotherapy (FLOT-protocol) were analyzed. As in the previous studies described, a major response of the primary tumor was seen more often in the radiochemotherapy group (17/40 pts. 43%) vs in the perioperative chemotherapy-group (11/40 pts. 27%) [23]. As in the previous Dutch study, no significant difference in survival between the two groups was found, and no comment was made regarding postoperative complications.

In summary of the three previous retrospective trials comparing the outcome of perioperative chemotherapy with neoadjuvant chemoradiation, two trials showed no advantage for one of the therapies and one trial showed a significant survival advantage for patients receiving perioperative chemotherapy. The results of our study seem to underline that neoadjuvant radiochemotherapy is not the treatment of choice for patients with AEG I.

Therefore, with the currently available data and as long as the results of the ongoing prospective trials are outstanding, we would not recommend CROSS as neoadjuvant treatment for patients with resectable adenocarcinoma of the distal esophagus.

Yet, the results of our trial also confirm the superior tumor tissue response in patients who received neoadjuvant chemoradiotherapy compared to chemotherapy. Therefore, we believe that regardless of the results of the ongoing prospective trials comparing CROSS with FLOT, a phase II study assessing the feasibility and safety of induction chemotherapy with FLOT followed by chemoradiotherapy with CROSS for locally advanced AEG I would be attractive. This treatment strategy would combine the local treatment impact of radiotherapy with the systemic control of chemotherapy and thus possibly lead to better survival. In any case, a neoadjuvant treatment concept seems reasonable since the majority of patients being treated with perioperative chemotherapy do not receive the adjuvant chemotherapy after surgery. In case of this study, only 39% of the patients received all postoperative cycles of chemotherapy, which is comparable to recent reports, but then again makes the superior outcome of the EOX group even more remarkable [22].

As an indicator of the quality of the esophagectomy and independent predictor of survival, the number of removed lymph nodes during surgery is considered [24]. Regardless of the surgical approach, the extent of lymphadenectomy during esophagectomy should be sufficient as it influences the survival of the patient. To maximize the survival benefit, a minimum of 23 regional lymph nodes must be removed [25, 26]. The standard actual is a two-field lymph node dissection abdominal and thoracic according to the German guidelines, which was performed in all cases in this study. A recent study evaluating the relation of neoadjuvant therapy to lymphadenectomy suggested that after neoadjuvant therapy, the expected lymph node yield should be 25% lower, and 32% lower after neoadjuvant chemoradiotherapy than after surgery alone [27]. A significant difference in the number of resected lymph nodes between the two therapies could be found in the current study. After CROSS therapy, the mean number of resected lymph nodes was 22 vs 29 after EOX. The observation that over 30% more lymph nodes were resected after chemotherapy indicates that the difference of the expected lymph node yield between chemotherapy and chemoradiotherapy might be even larger. The fact that the neoadjuvant treatment has a significant influence on the number of lymph nodes resected has some potential clinical impact and should be considered in guidelines and recommendations concerning lymph node dissection.

Strengths of this study are that it is a multicenter study representing the clinical reality of four different centers of one country. Furthermore, all patients included had AEG I and underwent curative esophagectomy, and in particular, this is the first trial comparing perioperative EOX vs neoadjuvant CROSS. Its retrospective character, as well as the lack of randomization and the inclusion of two groups receiving treatment in different time periods, is the limitation of this study.

In conclusion, there seem to be clear advantages for chemoradiation, concerning the major response of the primary tumor, whereas a tendency in favor for chemotherapy is seen in regard to systemic tumor control. Furthermore, the type of neoadjuvant treatment has a significant influence on the number of lymph nodes resected.

## Data Availability

The datasets that were analyzed during the current study are available from the corresponding author upon reasonable request.

## Abbreviations

18F-FDG PET: 18F-fluorodeoxyglucose positron emission tomography
AEG type I: Adenocarcinoma of the distal esophagus
AEG type II: Adenocarcinoma of the gastroesophageal junction
AEG type III: Adenocarcinoma of the proximal stomach
CROSS: Carboplatin/paclitaxel + RTX 41.4 Gy
CT: Computed tomography
DFS: Disease-free survival
EOX: Epirubicin, Oxaliplatin, Xeloda
FLOT: 5-Fluoruracil/leucovorin, oxaliplatin, and docetaxel
GOJ: Gastro-esophageal junction
OS: Overall survival
pCR: Pathological complete response
RTX: Radiotherapy
